# Effect of the reinforcement rate on goal-directed and habitual choices in a multiple schedule

**DOI:** 10.3389/fpsyg.2025.1601901

**Published:** 2025-07-02

**Authors:** Ting Hu, Shun Fujimaki, Hiroto Kawarada, Yutaka Kosaki

**Affiliations:** ^1^Department of Psychology, Waseda University, Tokyo, Japan; ^2^Research Institute for Letters, Arts and Sciences, Waseda University, Tokyo, Japan; ^3^Department of Psychology, Tokiwa University, Mito, Ibaraki, Japan

**Keywords:** operant (instrumental) conditioning, habitual, goal-directed, forced-choice training, reinforcement rate, multiple schedule of reinforcement, interval schedule, rats

## Abstract

Voluntary behaviors can be either goal-directed, sensitive to changes in their consequences, or habitual, lacking such sensitivity. In this study, we conducted three experiments to investigate how forced-choice training influences goal-directed and habitual processes under varying reinforcement rates. In all experiments, rats received 15 training sessions on a two-component multiple schedule with two sequentially inserted levers. In Experiment 1, identical variable interval (VI) 15-s schedules were used across components for Group Rich and VI 90-s schedules for Group Lean, yielding different behavioral outcomes. Following taste aversion for one outcome, Group Lean reduced performance (i.e., goal-directed action) during an extinction test, while Group Rich did not (i.e., habit). Experiment 2 addressed differential outcome exposure by reversing training conditions: Group Rich received numerous outcomes equivalent to Group Lean in Experiment 1, and vice versa. The devaluation effects were evident in both groups. Using the same outcome across components, Experiment 3 trained rats on a multiple VI 15-s VI 90-s schedule to further clarify the role of response–outcome pairings while controlling for the total amount of outcome exposure. Although the VI 15-s component produced fewer outcomes, it led to stronger devaluation effects and residual responding. The most important finding of this study is that alternating R–O contingencies in a multiple schedule under lean reinforcement conditions consistently sustain goal-directed control even after extensive training, while richer conditions promote a shift to habitual control. These findings are discussed within a dual-system model framework in a molar context, hypothesizing that both goal-directed and habitual strength may grow more rapidly with higher reinforcement rates.

## 1 Introduction

Contemporary learning theories emphasize the binary associative structure underpinning voluntary behaviors, mediated by either *Response-Outcome* (R-O) associations or *Stimulus–Response* (S-R) associations (Dickinson, 1985; Lingawi et al., 2016). Behaviors motivated by R-O associations are flexible and goal-directed, allowing modifications in response to changes in outcomes’ incentive value and R-O contingencies. Conversely, habits governed by S-R associations demonstrate persistence, lacking such sensitivities. Methodologies such as satiation, conditioned taste aversion, and contingency degradation have proven efficacious in discerning habits from goal-directed actions (Lingawi et al., 2016). Alterations in perceived value of the outcome achieved offline, for example, through pre-feeding the animals with the training outcome or pairing it with nausea-inducing drugs before testing under extinction, demonstrate goal-directed actions’ adaptability. Specifically, a noticeable performance decrease is indicative of goal-directed action, while a habit remains unaffected.

The amount of training and the specific reinforcement schedules used for training are important factors in determining whether a response is habitual or goal-directed. As responses are repeatedly performed in a consistent context, they gradually transition from being goal-directed to habitual after extended training (Adams, 1982; Dickinson et al., 1995; Holland, 2004; Fujimaki et al., 2024). Interval reinforcement schedules are typically more conducive to forming habits than ratio schedules when the outcome probabilities or training amount are matched (Dickinson et al., 1983; Gremel and Costa, 2013). According to the correlation view, a well-established hypothesis, the likelihood of a behavior being goal-directed is determined by the correlation between the response rate and outcome rate (Dickinson, 1985; Perez and Dickinson, 2020). Strong correlations favor goal-directed actions by strengthening R-O associations, whereas weak correlations promote habitual responses through dominant S-R associations. Within this theoretical framework, extensive training often reduces the variability of response rates, thereby decreasing the moment-to-moment correlation over time. The nature of the training schedule also significantly affects the dominance of S-R association versus R-O association. Ratio schedules, where outcomes are provided after a certain number of responses have been emitted, create a positive correlation between response rate and outcome rate, fostering goal-directed control. By contrast, interval schedules, where responses are reinforced after a certain amount of time has elapsed, do not make the response as strongly tied to its contiguous outcome and thus lean toward habitual control.

Contrary to common belief, interval schedules and extensive training do not necessarily lead to habitual learning; instead, providing a choice between two responses during the training or test sustains goal-directed control (Colwill and Rescorla, 1985, 1988; Kosaki and Dickinson, 2010). In a simultaneous choice procedure (Kosaki and Dickinson, 2010), rats subjected to random interval (RI) 60-s schedules on two concurrently available levers dispensing distinct outcomes exhibited goal-directed actions even after extensive training. This contrasts with the habits observed in rats trained to press a single lever for a specific outcome while receiving the other outcome non-contingently (Kosaki and Dickinson, 2010).

Animals encounter choices either simultaneously or sequentially. If habits are difficult to form under these conditions, it raises the question of whether predominant goal-directed control can be sustained in forced-choice scenarios or whether such control is exclusive to free-choice scenarios. Even though both scenarios include periods where animals engage with one contingency but not the other, only free-choice scenarios can include time samples with switches to alternative reinforcements, actively introducing variability for both responses. Surprisingly, little is currently known about the impact of forced-choice scenarios on habitual and goal-directed control, prompting questions about the necessity of active decision-making in maintaining this control. A relevant insight comes from a study demonstrating that habits develop readily when external stimuli guide choice (Turner and Balleine, 2024). Using a discrete-trial choice task, where discriminative stimuli biased choice toward the specific response delivering the outcome, choice between two concurrently accessible levers could be insensitive to outcome devaluation while showing stimulus–response specificity (Turner and Balleine, 2024). It appeared that externally triggered behavior, akin to a forced choice scenario, easily encourages the development of habitual control.

Before drawing conclusions, a noteworthy aspect is that the goal-directed choice in the simultaneous choice procedure (Kosaki and Dickinson, 2010) was demonstrated using a free-operant procedure without any discriminative stimuli except the response manipulanda. Therefore, an alternative view is that habitual choice develops because discrete R-O associations are quickly overridden by discrete S-R associations when salient stimuli, such as lights or tones, are used during two-lever training. Indeed, several studies have demonstrated that the presence of such stimuli promotes behavior insensitive to outcome devaluation under both single (Thrailkill et al., 2018; Thrailkill et al., 2021; Vandaele et al., 2017) and multiple R-O contingencies (Faure et al., 2005; Vandaele et al., 2020; Vandaele and Ahmed, 2021). Discriminative stimuli that reliably predict outcome availability appear to facilitate habit formation, perhaps by mitigating demands on attention to the response (Thrailkill et al., 2018). Additionally, reducing the predictability of outcome-associated stimuli has been shown to restore goal-directed control (Thrailkill et al., 2018; Thrailkill et al., 2021; Turner and Balleine, 2024).

Beyond choice training, the reinforcement rate also influences the dominance of R-O associations in interval schedules with extensive training (Garr et al., 2020). In one such experiment (Garr et al., 2020), lever-pressing exhibited sensitivity to outcome devaluation under an RI 15-s schedule rather than under an RI 45-s schedule after moderate training. With prolonged training, however, even the RI 45-s schedule contributed to goal-directed actions. To accommodate these results, it has been proposed that reinforcement rate affects the emergence of goal-directed control, with slower R-O association development on leaner RI schedules due to poorer R-O contiguity (Garr et al., 2020). Thus, performance on lean RI schedules may initially appear habitual after limited training but shift to goal-directed with extended training. Whether this pattern applies in choice tasks remains unclear.

The purposes of our experiments were twofold. First, since habits do not typically emerge when animals choose between simultaneously presented options (Kosaki and Dickinson, 2010), we examined whether active decision-making in selecting actions with different outcomes is a prerequisite for preventing S–R associations from becoming dominant. Considering that the discrete-trial choice procedure with salient stimuli effortlessly compels choices to become habitual, perhaps by reducing uncertainty about outcome availability (Faure et al., 2005; Thrailkill et al., 2018; Vandaele et al., 2020; Vandaele and Ahmed, 2021; Turner and Balleine, 2024), we investigated this question using a multiple reinforcement schedule, a procedure in which two or more reinforcement schedules alternate in a random sequence, each signaled by a distinct discriminative stimulus. In our case, however, no external cues other than the levers were used. We hypothesized that only weak S-R associations would form to compete with R-O associations in the current multiple reinforcement schedule, as the general behavioral context, rather than specific stimuli, became associated with both levers. This would hinder the rapid development of habitual control. If sequential R-O contingencies within the same behavioral context sustain goal-directed control, we anticipated observing sensitivity to outcome devaluation in this forced-choice procedure.

Second, we aimed to test the reproducibility and generality of previous findings (Garr et al., 2020) in choice settings, demonstrating that higher reinforcement rates promote the early emergence of goal-directed actions on interval schedules in single-lever tasks. If the strength of the R-O association develops at a faster rate and more robustly on rich interval schedules, as Garr et al. (2020) proposed, we expect to observe goal-directed choice in the rich VI schedules with higher reinforcement rates and habitual choice in the lean VI schedules with lower reinforcement rates.

## 2 Materials and methods

### 2.1 Subjects

Each of the three experiments had 24 experimentally naïve, male Wistar/ST rats purchased from Tokyo Laboratory Animals Science Co., Ltd., Japan (TLA). The rats were aged 9 to 10 weeks at the start of the experiment and were housed in groups of three animals in plastic cages (41 cm × 25 cm × 17 cm, length × width × height) in a temperature-controlled room that was on a 14:10 light–dark cycle (lights on at 08:00 a.m.). Experimental sessions were conducted during the light portion of the cycle at approximately the same time each day. Throughout the experiment, they were maintained at 85% of their free-feeding weights by daily supplemental feedings (given following the experiment session each day) with unrestricted access to water. All experiments were conducted in accordance with the Fundamental Guidelines for Proper Conduct of Animal Experiments and Related Activities in Academic Research Institutions and approved by the university’s ethical committee for animal experiments (Approval number: A24-149). All methods were carried out in accordance with relevant guidelines and regulations, including ARRIVE guidelines.

### 2.2 Apparatus

Twelve identical operant chambers (Med Associates, ENV-007CT), 32 cm long, 24.5 cm wide, and 29.5 cm high, were used. Each chamber was housed in a sound-attenuating box equipped with a ventilation fan. The sidewalls and ceilings of each chamber were made of Plexiglas, while the front and back walls were aluminum. Two retractable levers positioned 16 cm apart and 6.7 cm above the grid floor were located on the front wall. A 2.8-W lamp was positioned 8 cm above each lever. A force of approximately 0.25 N was required to operate each lever. Each chamber had a recessed food magazine in the center of the two levers. Attached to the food magazine was a pellet dispenser (Med Associates, ENV-203-45) delivering 45 mg food pellets (F0021; Bio-Serv, Flemington, NJ) into a small metal cup measuring 5 cm above the floor. A liquid drop dispenser (Med Associates, ENV-201A) was mounted behind the front panel that could deliver 0.1 mL drops of a 20% (w/v) sucrose solution containing 0.4% (v/v) vanilla flavoring (vanilla-sucrose solution) into the metal cup. A 3.3-W switchboard lamp mounted high on the back wall produced a steady houselight. Outside the chamber, a white noise generator consistently provided a background noise measuring 66 dBA. Experimental events were controlled and recorded automatically by an Intel-based PC with MED-PC IV software and relays located in the same room.

### 2.3 Procedure

#### 2.3.1 Experiment 1

Experiment 1 was run in two identical replications with 12 rats for each replication (Table 1).

**Table 1:** Design of experiments 1 ~ 3.

Experiment	Group	Multiple schedule components and response-outcome assignments	Component duration	Total number of outcomes	Devaluation	Extinction test	Reacquisition test
Experiment 1	Rich	R1-O1:VI 15 s; R2-O2: VI 15 s	5 min.	1,400	O1/O2-LiCl;O2/O1-NaCl	R1 vs. R2 (mult.)	R1-O1/R2-O2	Lean	R1-O1:VI 90 s; R2-O2: VI 90 s	5 min.	470
	Experiment 2	Rich	R1-O1:VI 15 s; R2-O2: VI 15 s	1 min.	470			O1/O2-LiCl;O2/O1-NaCl	R1 vs. R2 (mult.)	R1-O1/R2-O2	Lean	R1-O1:VI 90 s; R2-O2: VI 90 s	24 min.	1,400
Experiment 3		Devalued	R1-O1:VI 15 s; R2-O1: VI 90 s	R1: 1 min.R2: 24 min.	R1: 235R2: 700	O1-LiCl;	R1 vs. R2 (mult.)	R1-O1/R2-O1			Valued	O1-∅

Mult, multiple reinforcement schedule; R1/R2, left or right lever; O1/O2, food pellet or sucrose solution; LiCl, lithium chloride; NaCl, sodium chloride; ∅, no injection (group valued received LiCl injections on no-pellet day).

##### 2.3.1.1 Pretraining

All rats underwent two initial 30-min magazine training sessions in which 45 mg food pellets or 0.1 mL vanilla-sucrose solutions were delivered on a variable time (VT) 60-s schedule, with both levers retracted. Each session involved only one type of outcome, and the sequence of outcome types was counterbalanced across sessions within the same day. The rats were then trained to press two levers in separate sessions using a continuous reinforcement (CRF) schedule. Each lever was associated with a specific outcome type, with reinforcement continuing until 30 outcomes were delivered. The lever and outcome type assignments were counterbalanced across the subjects.

##### 2.3.1.2 Operant training

After pretraining, subjects were exposed to a two-component multiple schedule in which the same VI schedules were in effect. For Group Rich, the VI 15-s schedule was maintained in both components across 15 training days. For Group Lean, the VI values increased progressively: 15-s for the first two days, 45-s for the next two days, and 90-s for the remaining days. In the multiple schedule, food pellets were delivered during one component (food component), and vanilla-sucrose solutions were delivered during the other component (liquid component). All VI schedules comprised 12 intervals selected without replacement and constructed by Fleshler and Hoffman (1962) so that the probability of reinforcement remains constant across time.

All components were signaled by the illumination of the houselight and the insertion of the corresponding lever. The lever and outcome type assignments in the components were the same as during pretraining. Each component was 5 min long and was separated by a 1-min intercomponent interval (ICI) during which the lever was retracted and the houselight was turned off. The components were strictly alternated, with no more than two of the same presented in sequence, and each component was presented 3 times per session. Sessions began with a 1-min blackout before the first component and ended after a total of 6 components were presented. The food or liquid component was chosen pseudorandomly across the rats (counterbalanced) following the initial blackout and fixed across training days.

##### 2.3.1.3 Outcome devaluation

Following operant training, one outcome was devalued through taste aversion, with half the rats receiving devaluation with food pellets and the other half with sucrose solution (counterbalanced), ensuring matched performance on lever presses for devalued and valued outcomes based on the final training session.

Outcome devaluation consisted of five 2-day cycles. On 1 day of the first cycle, animals were placed in individual holding boxes (identical to home cages) in a separate feeding room from the test location and given unrestricted access to either food pellets or vanilla-sucrose solution (O1) for 15 min. Immediately thereafter, half of the rats received intraperitoneal injections of 0.15 M lithium chloride (LiCl) at 20 mL/kg, whereas the other half received saline injections before returning to their home cages. On the alternate day, rats were exposed to the other type of outcome (O2) for 15 min. Rats that received LiCl with O1 the day before were given saline injections, while those that previously had saline with O1 received LiCl injections.

The next four cycles were carried out in the operant chambers. In daily devaluation sessions, rats were placed in the chambers for 15 min with the levers retracted and were presented with either O1 or O2 delivered on a VT 60-s schedule. Rats received LiCl injections when presented with the devalued outcome and saline injections on days with the non-devalued outcome. Subsequently, they were returned to their home cages.

##### 2.3.1.4 Extinction test

All subjects received one test session under extinction conditions. The procedure was identical to that used during the operant training phase except that the responses were recorded without any programmed consequences.

##### 2.3.1.5 Reacquisition test

To prevent any reduction in performance for valued outcomes, it is essential to avoid delivering both valued and devalued outcomes in the same magazine. Therefore, rats were given two reacquisition tests on successive days, with only one lever inserted for 15 min. For Group Rich, responses were reinforced again on the VI 15-s schedule, whereas for Group Lean, responses were reinforced on the VI 90-s schedule. Each test began with a 1-min blackout during which all stimuli were turned off.

#### 2.3.2 Experiment 2

Experiment 2 was identical to Experiment 1 except for one procedural change in the operant training phase. In Experiment 1, we calculated the average number of outcomes delivered per group per session and then divided this by 6 (the total number of components) to set the component end time for Experiment 2. Consequently, rats on the multiple VI 15-s VI 15-s schedule received approximately 40 outcomes per lever for the first 2 days, then 18 for the next 2 days, and 12 thereafter. On the other hand, rats on the multiple VI 90-s VI 90-s schedule received about 40 outcomes per lever for the first 4 days and 50 for the remaining days. The components ended once the obtained number of outcomes met this criterion. As in Experiment 1, each component was presented three times per session. Unlike Experiment 1, however, Experiment 2 was conducted only once. All other experimental parameters and protocol details remained consistent with those in Experiment 1.

#### 2.3.3 Experiment 3

##### 2.3.3.1 Pretraining

All rats completed a single 30-min session of magazine training, receiving 45 mg food pellets on a VT 60-s schedule. This was followed by two sessions of lever-press training on a CRF schedule, training left and right levers in separate sessions, each concluding once rats achieved 30 outcomes.

##### 2.3.3.2 Operant training

After pretraining, subjects were exposed to a two-component multiple schedule that varied in reinforcement rate. In the component with a higher reinforcement rate (rich), rats earned food pellets according to a VI 15-s schedule maintained consistently over 15 sessions. In the component with a lower reinforcement rate (lean), the schedule began with VI 15-s for two sessions, changed to VI 45-s for the next two sessions, and finally transitioned to VI 90-s for the remaining 11 sessions. Each day, the number of outcomes delivered in the Rich component equaled half of the average achieved by the Group Lean of Experiment 1, while the Lean component similarly received half of the average obtained by the Group Rich of Experiment 1. Other experimental parameters and procedural details were the same as those used in previous experiments. Experiment 3 was conducted only once.

##### 2.3.3.3 Outcome devaluation

Following operant training, the rats were assigned to either the Devalued or Valued Group, matched for performance based on lever presses during the rich and lean components of the final training session. The outcome devaluation process was carried out over five 2-day cycles. On 1 day of the first cycle, rats were placed in individual boxes with free access to food pellets for 15 min. Those in Group Devalued then received an immediate intraperitoneal injection of 20 mL/kg LiCl (0.15 M), while those in Group Valued were returned to home cages without an injection. On the alternate day, rats were placed in boxes without food pellets for 15 min. Following this, Group Valued rats received LiCl injections, whereas Group Devalued rats did not.

The subsequent four cycles were conducted in the operant chambers. In each cycle, rats received either 15 food pellets on a VT 60-s schedule 1 day or mere exposure to the chamber for the same duration the other day. Based on the group assignments, LiCl injections were administered to devalued rats only on pellet-receiving days and to valued rats only on no-pellet days. During the conditioning sessions, the houselight was turned on without the presentation of levers.

##### 2.3.3.4 Extinction test

All rats underwent one extinction test, the same as in Experiment 1. Each component was 5 min long, presented 3 times, and separated by a 1-min ICI. The test began with a 1-min blackout and concluded after a total of 6 components were presented. No outcomes were delivered during the test.

##### 2.3.3.5 Reacquisition test

The left and right levers were tested in separate 15-min sessions. The lever used during the Rich component was reinforced again on a VI 15-s schedule, whereas the lever from the Lean component was reinforced on a VI 90-s schedule. Each session started with a 1-min blackout, with the order of lever presentation remaining consistent in the operant training phase.

### 2.4 Data analysis

The primary dependent variables were the proportion of baseline and absolute response rates. The data were subjected to mixed-design analyses of variance (ANOVAs), followed by *post hoc* comparisons when indicated. The rejection criterion was set to 0.05 for all statistical tests. When Mendoza’s test indicated a violation of sphericity, the Greenhouse–Geisser correction was applied to the degrees of freedom. *Post hoc* multiple comparisons for ANOVAs were conducted using Shaffer’s modified Bonferroni procedure (Shaffer, 1986). Effect sizes were calculated for all significant effects using generalized eta-squared (*η_G_^2^*; Bakeman, 2005; Olejnik and Algina, 2003) and interpreted according to conventional guidelines for interpreting η^2^ (Cohen, 1988), with *η_G_^2^* values of approximately 0.02, 0.13, and 0.26 considered indicative of small, medium, and large effects, respectively. Planned comparisons were conducted within the ANOVA framework and evaluated using F-tests. Post hoc power analyses were performed to assess the adequacy of the sample size for Experiment 1. Data were analyzed using RStudio, version 2024.09.0 + 375.

## 3 Results

### 3.1 Experiment 1

#### 3.1.1 Operant training

**Figure 1:**
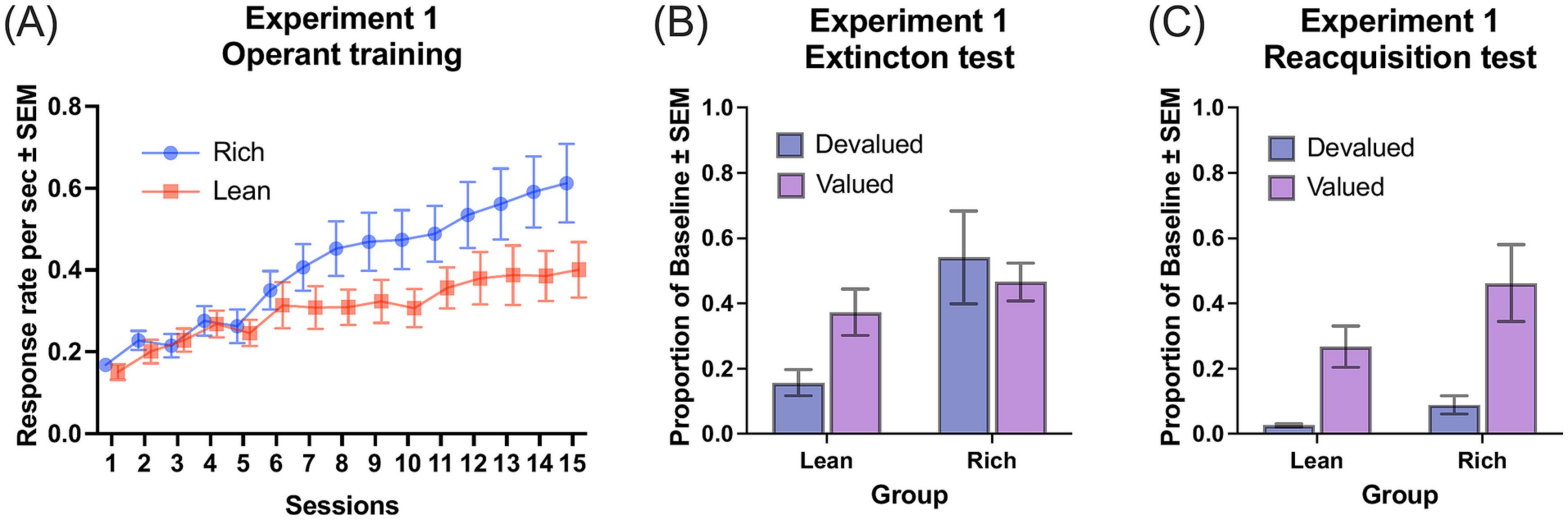
Performance in experiment 1. **(A)** Response rates across operant training sessions. Group Rich: multiple VI 15-s VI 15-s schedule with 1,400 outcomes; Group Lean: multiple VI 90-s VI 90-s with 470 outcomes. **(B)** Proportion of baseline response rates during the extinction test following outcome devaluation. **(C)** Proportion of baseline response rates during the reacquisition test. Error bars represent SEM.

Figure 1A illustrates the acquisition of discriminated responses occasioned by different levers during the operant training. A Group (rich and lean) × Session (15) ANOVA revealed a significant main effect of Session, *F*(14, 308) = 31.75, *MSE* = 0.28, *p* < 0.001, *η_G_^2^* = 0.32, with sufficient statistical power (1−*β* > 0.99). Though Group Rich exhibited only marginally higher response rates than Group Lean, *F*(1, 22) = 3.51, *p* = 0.07, *η_G_^2^* = 0.10, a significant Group × Session interaction was observed, *F*(14, 308) = 4.07, *MSE* = 0.04, *p* < 0.001, *η_G_^2^* = 0.06. Simple main effects analysis indicated that lever pressing increased reliably over sessions for both groups, smallest *F*(14, 154) = 9.73, *MSE* = 0.07, *p* < 0.001, *η_G_^2^* = 0.21. Response rates differed significantly between groups at Sessions 8, 10, 14 and 15, smallest *F*(1, 22) = 4.72, *MSE* = 0.27, *p* = 0.04, *η_G_^2^* = 0.18.

#### 3.1.2 Extinction test

Figure 1B summarizes the critical data from the extinction test. Given the varying baseline response rates between the rich and lean groups, performance during the extinction test was normalized to baseline proportions. A Devaluation (valued and devalued) × Group (rich and lean) ANOVA on proportions of baseline response rates revealed a significant main effect of Group, *F*(1, 22) = 6.78, *MSE* = 0.68, *p* = 0.02, *η_G_^2^* = 0.15, indicating that the Group Rich pressed more than the Group Lean overall. *Post hoc* power analysis indicated that the achieved power was approximately 0.82. A marginal devaluation × group interaction was observed, *F*(1, 22) = 3.16, *p* = 0.09, *η_G_^2^* = 0.06, and the main effect of Devaluation was not significant, *F*(1, 22) = 0.73, *p* = 0.40. To test our *a priori* hypothesis, planned comparisons were conducted within and between groups. Within-group comparisons revealed that Group Lean rats showed a significant difference between responses to the devalued and valued outcomes, *F*(1, 11) = 19.13, *MSE* = 0.28, *p* < 0.01, *η_G_^2^* = 0.24, whereas Group Rich rats showed no significant difference, *F*(1, 11) = 0.23, *p* = 0.64. Planned comparisons between groups further revealed that Group Lean and Group Rich rats differed significantly for the devalued outcome, *F*(1, 22) = 6.76, *MSE* = 0.89, *p* = 0.02, *η_G_^2^* = 0.24, but not for the valued outcome, *F*(1, 22) = 1.03, *p* = 0.32.

#### 3.1.3 Reacquisition test

Figure 1C shows the data from the reacquisition test. A Devaluation × Group ANOVA revealed a reliable main effect of Devaluation, *F*(1, 22) = 17.95, *MSE* = 1.13, *p* < 0.001, *η_G_^2^* = 0.31, with sufficient statistical power (1−*β* > 0.99). Both rich and lean groups pressed less on the lever associated with the devalued outcome than the lever associated with the valued outcome, indicating successful devaluation. Although there was a trend suggesting that the Group Rich responded more than the Group Lean, *F*(1, 22) = 3.99, *p* = 0.058, *η_G_^2^* = 0.07, the Devaluation × Group interaction did not approach significance, *F*(1, 22) = 0.83, *p* = 0.37.

### 3.2 Experiment 2

#### 3.2.1 Operant training

**Figure 2:**
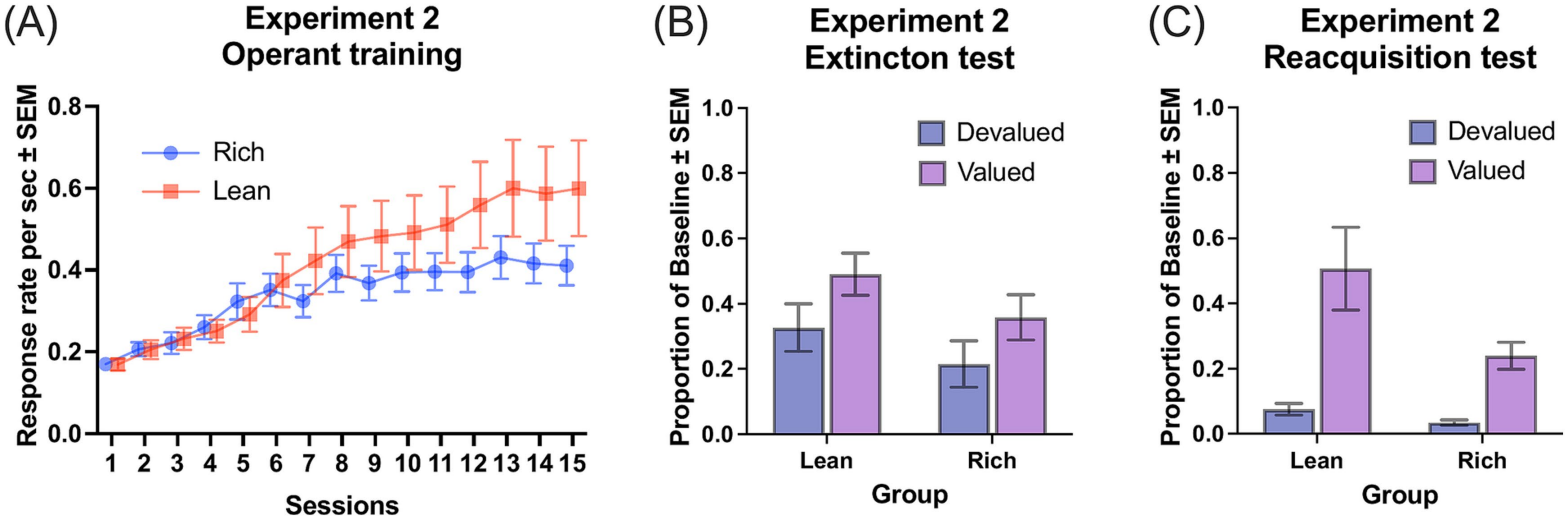
Performance of experiment 2. **(A)** Response rates across operant training sessions. Group Rich: multiple VI 15-s VI 15-s schedule with 470 outcomes; Group Lean: multiple VI 90-s VI 90-s with 1,400 outcomes. **(B)** Proportion of baseline response rates during the extinction test following outcome devaluation. **(C)** Proportion of baseline response rates during the reacquisition test. Error bars represent SEM.

Figure 2A shows the acquisition of discriminated responses signaled by different levers across training sessions. A Group × Session ANOVA revealed a significant main effect of Session, *F*(14, 308) = 37.12, *MSE* = 0.33, *p* < 0.001, *η_G_^2^* = 0.40 and a significant Group × Session interaction, *F*(14, 308) = 3.88, *MSE* = 0.03, *p* < 0.001, *η_G_^2^* = 0.07. The overall difference in lever pressing between groups was marginally significant, *F*(1, 22) = 3.05, *p* = 0.09, *η_G_^2^* = 0.08. Simple main effect analyses indicated significant group differences at Sessions 12, 13, 14 and 15, smallest *F*(1, 22) = 4.74, *MSE* = 0.17, *p* = 0.04, *η_G_^2^* = 0.18.

#### 3.2.2 Extinction test

Figure 2B demonstrates the extinction test data, assessing devaluation sensitivity as the proportions of baseline response rates. A Devaluation × Group ANOVA showed a significant main effect of Devaluation, *F*(1, 22) = 10.57, *p* = 0.004, *MSE* = 0.28, *η_G_^2^* = 0.10. No effects or interactions involving Group approached significance, largest *F*(1, 22) = 2.00, *p* = 0.17.

#### 3.2.3 Reacquisition test

Figure 2C displays the response rates during the reacquisition test. A Devaluation × Group ANOVA showed significant main effects of Devaluation, *F*(1, 22) = 21.47, *MSE* = 1.21, *p* < 0.001, *η_G_^2^* = 0.34, and Group, *F*(1, 22) = 5.38, *MSE* = 0.28, *p* = 0.03, *η_G_^2^* = 0.11, indicating that Group Lean responded significantly more than Group Rich. There was no significant Devaluation × Group interaction, *F*(1, 22) = 2.72, *p* = 0.11.

### 3.3 Experiment 3

#### 3.3.1 Operant training

**Figure 3:**
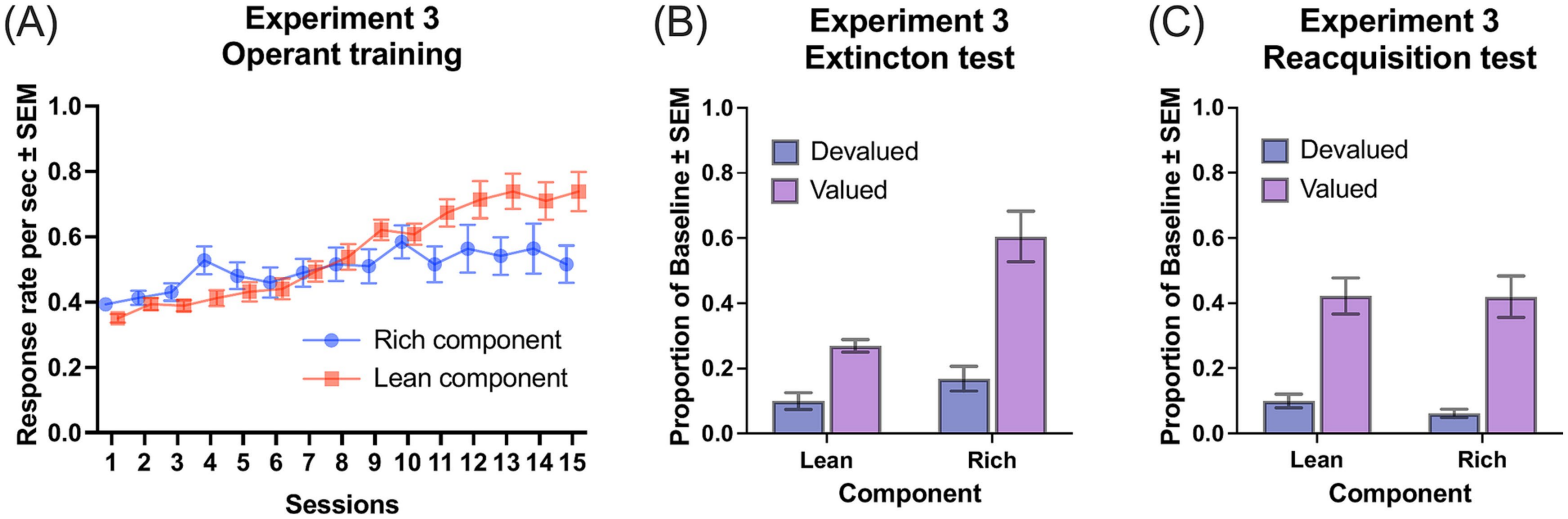
Performance in experiment 3. **(A)** Response rates across operant training sessions under a multiple VI 15-s VI 90-s schedule: rich component (VI 15-s, 235 outcomes) and lean component (VI 90-s, 700 outcomes), both delivering the same outcome (O1). **(B)** Proportion of baseline response rates during the extinction test following outcome devaluation. **(C)** Proportion of baseline response rates during the reacquisition test. Error bars represent SEM.

Figure 3A depicts the development of discriminative responses across the rich and lean components throughout the training sessions. A Session (15) × Component (rich and lean) ANOVA showed a significant main effect of Session, *F*(2.87, 65.91) = 16.97, *MSE* = 0.13, *p* < 0.001, *η_G_^2^* = 0.16. While the main effect of Component did not reach statistical significance, *F*(1, 23) = 2.83, *p* = 0.11, there was a significant Session × Component interaction, *F*(4.35, 99.95) = 9.47, *MSE* = 0.42, *p* < 0.001, *η_G_^2^* = 0.05. As training progressed, rats consistently pressed both levers, smallest *F*(4.05, 93.11) = 3.17, *MSE* = 0.08, *p* = 0.02, *η_G_^2^* = 0.05. The Lean component produced significantly lower response rates than the Rich component in sessions 1 and 4, smallest *F*(1, 23) = 9.19, *MSE* = 0.02, *p* = 0.006, *η_G_^2^* = 0.09, and significantly higher response rates in Sessions 9, 11, 12, 13 and 15, smallest *F*(1, 23) = 4.45, *MSE* = 0.06, *p* = 0.046, *η_G_^2^* = 0.05.

#### 3.3.2 Extinction test

Figure 3B presents the primary data from the extinction test, with devaluation sensitivity measured as a proportion of baseline response rates. A Group (devalued and valued) × Component (rich and lean) ANOVA showed significant main effects of Devaluation, *F*(1, 22) = 30.52, *MSE* = 1.10, *p* < 0.001, *η_G_^2^* = 0.49, and Component, *F*(1, 22) = 32.39, *MSE* = 0.49, *p* < 0.001, *η_G_^2^* = 0.30, as well as a Group × Component interaction, *F*(1, 22) = 14.11, *MSE* = 0.21, *p* = 0.001, *η_G_^2^* = 0.16. Simple main effect analyses revealed that rats in Group Devalued made significantly fewer responses than those in Group Valued during both the Rich component, *F*(1, 22) = 25.40, *MSE* = 1.14, *p* < 0.001, *η_G_^2^* = 0.54, and Lean component, *F*(1, 22) = 27.62, *MSE* = 0.17, *p* < 0.001, *η_G_^2^* = 0.56. Furthermore, the reduction in response rates from the baseline was significantly smaller in the Rich component than in the Lean component for both the Group Valued, *F*(1, 11) = 25.77, *MSE* = 0.67, *p* < 0.001, *η_G_^2^* = 0.44, and the Group Devalued, *F*(1, 11) = 6.98, *MSE* = 0.03, *p* = 0.02, *η_G_^2^* = 0.09.

#### 3.3.3 Reacquisition test

Figure 3C shows the response rates during the reacquisition test. A Group (devalued and valued) × Component (rich and lean) ANOVA revealed a significant main effect of Group, *F*(1, 22) = 45.49, *MSE* = 1.39, *p* < 0.001, *η_G_^2^* = 0.57. There were no significant main effects of component or devaluation × component interaction, largest *F*(1, 22) = 0.31, *p* = 0.58.

## 4 Discussion

The primary aims of our experiments were, first, to determine whether forced-choice training involving sequential presentation of two responses could maintain goal-directed control on interval schedules after extensive training and, second, to evaluate the impact of different reinforcement rates on the shift from goal-directed to habitual behaviors in choices. Initially, we trained rats using a two-component multiple schedule, in which two levers were presented sequentially across components, each signaling the same VI schedules but producing different outcomes (O1 and O2). In Experiment 1, we observed sensitivity to devaluation on the multiple VI 90-s VI 90-s schedule and devaluation insensitivity on the multiple VI 15-s VI 15-s schedule, suggesting that higher reinforcement rates may foster habit formation. However, controlling for component duration exposed the rats more extensively to outcomes under the richer VI schedules. To address the confounding effect of differential outcome exposure level on devaluation sensitivity, Experiment 2 equalized the total number of outcomes with those in Experiment 1 across groups, evaluating two conditions: one group received fewer outcomes under rich VI schedules (470 outcomes), whereas the other obtained more outcomes under lean VI schedules (1,400 outcomes). Both groups demonstrated significant devaluation effects, suggesting a dynamic interaction between goal-directed and habitual processes, concurrently influenced by reinforcement rate and cumulative experience of outcomes. The collective results from Experiments 1 and 2 indicate that higher reinforcement rates facilitate goal-directed behavior in the early stage, but habits can form with extensive outcome exposure. In contrast, lower reinforcement rates tend to always favor goal-directed control. Finally, Experiment 3 revisited the relationship between reinforcement rate and training amount, focusing on disentangling their contributions to devaluation resistance within the multiple schedule. In Experiment 1, higher reinforcement rates encouraged habitual behavior with 700 response-contingent outcomes; however, this effect diminished in Experiment 2 with 235 outcomes, leaving their relative influence uncertain. In Experiment 3, rats were trained on a multiple VI 15-s VI 90-s schedule, wherein the same outcome (O1 only) was delivered in greater total numbers in the lean component than in the rich one. The stronger devaluation effect observed in the rich component, which generated fewer outcomes, suggests that the training amount contributes more to resistance to devaluation than the reinforcement rate in our experiments. Experiment 3 also replicated the finding from Experiment 2, demonstrating that lower reinforcement rates preserved sensitivity to devaluation. This effect persists regardless of whether 235 or 700 outcomes were earned per response and whether cumulative outcomes reached 470, 935, or 1,400.

Our results both corroborate and contradict various findings in the literature. Our devaluation effects are consistent with those demonstrating that multiple R-O contingencies preserve goal-directed control over behavior, even after prolonged training on interval schedules (Colwill and Rescorla, 1985, 1988; Holland, 2004; Kosaki and Dickinson, 2010). Crucially, the Group Lean in Experiment 2 displayed goal-directed choice with approximately 700 response-outcome pairings of each response, far exceeding the typical numbers for habit formation observed in single R-O contingencies (Adams, 1982; Dickinson et al., 1995). However, this effect seems specific to the leaner reinforcement schedule, as similarly extensive training with two VI 15-s components in the multiple schedule led to habitual responding.

A major novel finding of our study is that goal-directed control persisted even when different responses were trained in alternating stimulus contexts, with the response manipulanda functioning as discriminative stimuli, irrespective of whether the contingent outcomes differed. Our devaluation effects closely resemble those documented for two different R-O contingencies trained in separate sessions (Colwill and Rescorla, 1985, 1988). According to dual-process theory, goal-directed learning relies on a short-term memory system calculating the current correlation between response rates and outcome rates across different time samples (Perez and Dickinson, 2020). While registered variations in response rates diminish over time under a single R-O contingency, additional sources of variation emerge as an inevitable consequence of training under two concurrent R-O contingencies. Short-term memory cycles involving changeover behaviors will register no response or outcome from the now non-engaged contingency and others with these representations from the engaged one. This explains the differing sensitivity to devaluation observed in concurrent-choice versus single-response training (Kosaki and Dickinson, 2010; Perez and Dickinson, 2020). However, the dual-process theory also has limitations in accounting for sustained goal-directed control under temporally separated R-O contingencies. Specifically, it posits that animals experience a sustained positive rate correlation only if the representations of the currently engaged response and its outcome, alongside the representations of their absence, are temporally adjacent within the same short-term memory cycle (Perez and Dickinson, 2020). To accommodate our devaluation findings and those of Colwill and Rescorla (1985, 1988), an increase in memory capacity must be assumed, allowing reinforcement-specific representations to be retrieved and integrated across temporally distinct episodes within a shared behavioral context.

At first glance, our results from Experiments 1 and 2 appear to contradict earlier research (Garr et al., 2020), which reported that habitual behaviors tend to emerge more rapidly on lean RI schedules. However, two key differences in experimental design may account for this discrepancy. First, although our interval schedules were even leaner, the cumulative number of outcomes provided was higher than in Garr et al. (2020) and thus not inconsistent with their suggestion that R–O associations develop more gradually under lean schedules and therefore require greater outcome exposure to detect goal-directed control. Accordingly, it remains possible that responding on VI 90-s components could have been insensitive to devaluation with more limited training involving fewer than 470 outcomes. Second, unlike Garr et al. (2020), who used a single reinforcement schedule, we employed a multiple reinforcement schedule with heterogeneous components. As discussed, such a forced-choice procedure involving multiple outcomes and operant contingencies appeared to maintain goal-directed control easily, even with extensive outcome exposure.

The devaluation effects observed in Experiment 3 revealed a complex interaction between reinforcement rate and response-outcome pairings in determining total response strength. Despite fewer outcomes, Group Devalued showed higher residual responding on the VI 15-s component versus the VI 90-s component within the same rats, suggesting that devaluation produced smaller reductions in response rate. The devaluation effect was also significantly stronger for the VI 15-s component than for the VI 90-s component when compared with Group Valued. This raises the question: how could higher reinforcement rates produce behavior that is simultaneously more sensitive and resistant to devaluation? By the final devaluation cycle, all rats had ceased consuming the pellets. This finding rules out the possibility that residual responding was due to incomplete devaluation. Given that devaluation-sensitive responding is inherently goal-directed, the residual responding observed after devaluation is, by necessity, more likely attributable to S-R habitual learning, as it remains unaffected by the current value of the outcome (Perez and Dickinson, 2020; Thrailkill and Bouton, 2015). If one accepts this proviso, a plausible explanation would be that both goal-directed and habitual response strength develop more rapidly on rich interval schedules. This leads to the next question: What mechanism underlies the influence of reinforcement rate on the balance between goal-directed and habitual control on interval schedules?

The proposed mechanism is that outcomes programmed to occur in closer temporal proximity on rich interval schedules may contribute to an early experience of a relatively strong rate correlation. Higher reinforcement rates may drive response rates to asymptote quickly, thereby accelerating the development of habit strength at the same time. During the early stage of training, responses are random and emitted regardless of reinforcement. Nevertheless, the likelihood of an outcome following a response is higher when shorter intervals are programmed between outcomes. This may cause the response-outcome rate feedback function to rise rapidly, leading to an early growth of goal-directed R-O association on rich schedules. In contrast, lean interval schedules exhibit a relatively weak feedback function, which grows slowly. Premature devaluation before animals detect a positive feedback function or perceive a causal relationship between response and reinforcement may render goal-directed action undetectable. However, as the response rates fall low enough to be optimal, a VI schedule increasingly resembles continuous reinforcement (Baum, 1973, 1981, 1992). At this extreme, the VI feedback function approximates that of continuous reinforcement, making goal-sensitive responding evident as VI performance stabilizes. This time window for lean interval schedules takes longer, allowing the optimal response rate to stabilize gradually. Once a response pattern governed by inter-response-time (IRT) reinforcement stabilizes, and the optimal rate reaches its upper limit, S-R associations may increasingly take primary control, with habit strength peaking faster on interval schedules reinforced by short IRTs than those with long IRTs. This framework efficiently explains all our findings and the previous observations (Garr et al., 2020) that responses on lean RI schedules exhibited devaluation sensitivity after extended training. Though previous research (Garr et al., 2020) implied that the probability of a response being goal-directed increases as a function of training amount, we argue this effect is temporary. If behavior is viewed as a continuous flow over an extended temporal framework (Baum, 1973; Baum and Aparicio, 2020; Baum, 2024), with more time or additional training, devaluation-sensitive responding is likely to revert to habitual status once optimal performance stabilizes.

Several additional comments merit further discussion. Prior studies hypothesized that good response-outcome contiguities foster an early emergence of goal-directed action in rich RI schedules, whereas poor contiguities bias habits on lean RI schedules (Garr et al., 2020; DeRusso et al., 2010). This contiguity-based learning rule could be integrated into the theoretical framework outlined above, where the effects of response-outcome contiguity operate through its influence on the correlation. Close response-outcome contiguity guarantees a strong correlation by minimizing variability around the feedback function (Baum, 1973). However, recognizing that the key factor in sustaining a response is the correlation between the responses and reinforcement, rather than the immediate contiguity, simplifies the understanding of operant conditioning (Baum, 1973; Baum and Aparicio, 2020). Poor contiguity may reduce response rates, but does not eliminate them as long as reinforcement continues. Perceived causal relationship or correlation is likely to strengthen as the VI responding approaches optimal performance. Considering that responses are related to reinforcement within a broader, more molar context, this explanation is not inconsistent with the findings of Garr et al. (2020).

Another unusual aspect is that the devaluation effects observed in Experiment 3 diverged from earlier studies (Holland, 2004), which demonstrated that extensive two-lever training maintained devaluation sensitivity only when multiple outcomes, rather than a single outcome, were used. Even with a single outcome, goal-directed control persisted in our study after extensive training. It should be noted that although Holland (2004) used longer training sessions, our rats received more cumulative reinforcers (950 vs. 810) under conditions that would typically favor habit formation. However, a key difference in our design is that, whereas Holland (2004) trained two responses with the same outcome and identical interval schedules, we trained two responses with the same outcome but different interval schedules. Our design may have contributed to more easily differentiated R–O associations, maintaining a high correlation rate and thereby sustaining goal-directed control.

Finally, our methodology aligns with standard practices in resistance-to-change research, which evaluates response strength under different reinforcement conditions when disrupted by prefeeding, response-independent events, or extinction. Arising from a distinct objective, however, our study is the first to investigate the effects of reinforcement rates in the multiple schedule using conditioned taste aversion as a disruptor. Experiment 3 corroborates prior findings that responses reinforced at higher reinforcement rates exhibit greater resistance to disruption (Grace and Nevin, 2000; Igaki and Sakagami, 2004; Nevin, 1974). However, Experiments 1 and 2 utilized different outcomes across components and between-subjects comparisons to examine reinforcement rate effects. Baseline variations across individuals and differential disruptions between food pellets and sucrose solutions (Podlesnik and Shahan, 2008) complicate direct comparisons, making it challenging to draw parallel conclusions.

One limitation of the present study is that all subjects were male rats. Although the use of a single sex controls for variability and is common in behavioral research, it limits the generalizability of the findings across sexes. Emerging evidence suggests that females develop habitual responding more rapidly than males under identical training conditions (Schoenberg et al., 2019). Future research should examine whether the patterns observed here extend to female subjects. In addition, the proposed interpretation that higher reinforcement rates may simultaneously promote both goal-directed and habitual strength remains speculative. While this framework is consistent with the current behavioral data, it has not been directly tested at the mechanistic level. Future neurobiological investigations are needed to evaluate this working hypothesis and identify the underlying processes supporting dual-system involvement under varying reinforcement conditions.

In summary, our forced-choice procedure, wherein animals exploit or dismiss single reinforcement opportunities sequentially, demonstrated that simultaneous choice training is not the exclusive method for maintaining goal-directed control after extensive training. In the multiple reinforcement schedules we utilized, two responses were either associated with the identical operant contingency but different outcomes (Experiments 1 and 2) or with different contingencies but the same outcome (Experiment 3). Habits were generally difficult to form under these conditions unless responses were reinforced by an exceptionally high number of outcomes at a high rate. Key findings include: (a) in Experiments 1 and 2, even with comparable numbers of outcomes, higher reinforcement rates initially promoted devaluation sensitivity, which diminished with prolonged exposure to outcomes, while lower reinforcement rates consistently supported goal-sensitive responding; (b) in Experiment 3, higher reinforcement rates resulted in a stronger devaluation effect and more robust residual responding, even with fewer total outcomes. These demonstrations, interpreted within the dual-system model (Perez and Dickinson, 2020), led us to assume that both goal-directed and habitual strengths accelerate more rapidly under higher reinforcement rates in interval schedules. Considering the feedback function as a continuous flow over time (Baum, 1973, 2024), we concur with prior studies that the expression of habitual and goal-directed behaviors is a dynamic process influenced by specific training conditions (Garr et al., 2020).

## Data Availability

The raw data supporting the conclusions of this article will be made available by the corresponding author, without undue reservation.
